# Phytochemical analysis and biological activities of solvent extracts and silver nanoparticles obtained from *Woodwardia unigemmata *(Makino) Nakai

**DOI:** 10.1371/journal.pone.0312567

**Published:** 2025-01-15

**Authors:** Syed Ahsan Shah, Alia Gul, Ghulam Mujtaba Shah, Maha Saeed Ibrahim Wizrah, Awais Khalid, Mamoona Munir, Zainab Maqbool, Arusa Aftab, Mazen R. Alrahili, Ayesha Siddiqua, M. Yasmin Begum

**Affiliations:** 1 Department of Botany, Hazara University, Mansehra, Khyber Pakhtunkhwa, Pakistan; 2 Department of Biology, College of Science and Humanities in Al-Kharj, Prince Sattam bin Abdulaziz University, Al-Kharj, Saudi Arabia; 3 Department of Physics, College of Science and Humanities in Al-Kharj, Prince Sattam bin Abdulaziz University, Al-Kharj, Saudi Arabia; 4 Department of Botany, Rawalpindi Women University, Rawalpindi, Pakistan; 5 Department of Botany, Lahore College for Women University, Lahore, Pakistan; 6 Physics Department, School of Science, Taibah University, Medinah, Saudi Arabia; 7 Department of Clinical Pharmacy, College of Pharmacy, King Khalid University, Abha, Saudi Arabia; 8 Department of Pharmaceutics, College of Pharmacy, King Khalid University, Abha, Saudi Arabia; Vietnam Academy of Science and Technology, VIET NAM

## Abstract

Multidrug resistant bacteria are causing health problems and economic burden worldwide; alternative treatment options such as natural products and nanoparticles have attained great attention recently. Therefore, we aimed to determine the phytochemicals, antibacterial potential, and anticancer activity of *W*. *unigemmata*. Extracts in different organic and inorganic solvents were prepared, silver nanoparticles were prepared using the green synthesis method. Phytochemicals and antioxidant activity was determined spectrophotometry, anticancer potential was determined against gastric cancer and normal gastric epithelial cells using CCK8 and colony formation assays *W*. *unigemmata* was found to have a significant enrichment of various phytochemicals including flavonoids, terpenoids, alkaloids, carotenoids, tannins, saponins, quinines, carbohydrates, phenols, coumarins and phlobatanins. Among them phenolics (5289.89 ± 112.67) had high enrichment followed by reducing sugar (851.53 ± 120.15), flavonoids (408.28 ± 20.26) and ascorbic acid (347.64 ± 16.32), respectively. The extracts prepared in organic solvents showed strong antibacterial activity against *P*. *aeruginosa* (chloroform, 13.66±0.88, ethyl acetate, 8.66±4.33, methyl alcohol, 13.33±1.66, N-hexane, 12.33±0.88) and *S*. *aureus* (chloroform, 15±0.57, ethyl acetate, 16.33±0.33, methyl alcohol, 17.66±0.33 and N-hexane, 16.33±0.33). Aqueously prepared AgNPs showed remarkable activity against *P*. *aeruginosa* follwed by *E*. *coli*, 17.66 ± 1.85, *S*. *aureus*, 16.00 ± 1.73, *K*. *pneumoniae*, 14.33 ± 1.20, respectively. The ethanolic extracts (500 μg, 1000 μg, 2000 μg) of the *W*. *unigemmata* were found to have cytotoxicity against both gastric cancer (AGS and SGC7901) and normal cell lines (GES-1); a significant cellular proliferation arrest was observed.

These results suggest that *W*. *Unigemmata* contains numerous bioactive phytochemicals and can be useful as a drug against MDR bacterial strains. These biomolecules covering AgNPs may enhance their biological activities, which can be employed in the treatment of various microbial infections.

## Introduction

Nanoparticles, due to tiny size, shape and morphology have ability to potentially interact with different cellular components of the animals, plants, and microbes; interaction usually governed by high surface-to-volume ratio (HSV), surface plasmon resonance (SPR), and surface-enhanced Raman scattering (SERS) [1]. Nowadays, nanomaterials synthesized from various sources are being used in our daily life including Zinc oxide NPs are used as a disinfectant [2], and gold nanoparticles against diagnosis of influenza virus [3], titanium oxide and silver NPs as antimicrobial [4]. Furthermore, AgNPs have been effectively used for clinical diagnosis and treatment of various diseases due to their cytotoxic, biolabeling and better drug delivery capability [5]. AgNPs synthesized from various plant extracts have shown strong antioxidant, antibiofilm, anticancer, antibacterial, and other biological activities [6]. Similarly, AgNPs due to their various valuable optical characteristics have high applications in biological and chemical sciences [7]. Due to extensive use of antibiotics and other drugs microbes are getting smarter, they have developed bypass mechanisms to survive in harsh drug environment [8], thus causing a serious health and economic crisis [9, 10]. Thus, commonly used antibiotics are no longer effective against such strains; warranting alternative treatment options such as natural products and nanomaterials [11].

Recently, the correlation with chronic infections and various cancers have been identified and cancer is growing rapidly and ranked as leading cause of death worldwide [12], only in 2018 about 9.6 million patients died due to cancer [13]. Gastric cancer (GC) is the fourth most common type of cancer that is present in the human population worldwide and among all cancers, the second highest mortality is caused by gastric cancer [14]. According to epidemiological data from the International Agency for Research on Cancer (IARC), there were 1,089,103 new cases of GC in 2020, with an estimated age-standardized incidence rate of 11.1% worldwide [15]. Among traditional anticancer medicines about 50% of approved anticancer drugs are obtained from plants such as irinotecan, paclitaxel, etoposide, vincristine, etc. [16]. Therefore, it is suggested that medicinal plants can be used as alternative drugs or supplemented with regular anticancer drugs [17]. Historically, natural product-based medicines are utilized by all civilizations as a traditional remedy to cure various diseases, and currently plants are considered a home for the discovery of novel drugs [18]. Among vascular plants, the use of pteridophytes (ferns) in folk medicine is well known. Pteridophytes are used as an effective remedy for disease management by various systems of medicines like Ayurvedic, Unani and homeopathic medicine [19]. Various secondary metabolites and morphological characteristics of pteridophytes help them tolerate the ruthless circumstances of terrestrial surroundings, and some of these phytochemicals can be used against pathogens [20]. Various biologically active components isolated from pteridophyte extracts such as steroids, phenolics, flavonoids, etc. have variety of medicinal properties [21]. *W*. *unigemmata* has significant ethano medicinal values. Fronds and rhizomes decoction of this plant used in dysentery, fronds used in infertility and skin diseases, dry rhizome used as purgative [22]. Potent polyphenols are extracted from the *W*. *unigemmata* extract showing remarkable antioxidant and antibacterial potential [23].

In the current study, we applied a green synthesis method of preparing AgNPs using an extract from *W*. *unigemmata*. We aimed to explore its potential as a green and sustainable source to produce AgNPs. Furthermore, we investigated the antibacterial efficacy of the nanoparticles. Furthermore, we also analyzed the phytochemicals, antioxidants and cytotoxicity of the *W*. *unigemmata* extracts. This groundbreaking research fills an important gap in current knowledge to utilize *W*. *unigemmata* in nanotechnology and biomedical research.

## Materials and methods

### Plant collection

Whole plant *W*. *unigemmata* was collected from District Mansehra, KPK, Pakistan, identified and processed at the Department of Botany Hazara University Mansehra. The plant was submitted in the Hazara University herbarium under voucher specimen number, Ali Gul and Syed Ahsan Shah 2061(HUP); Mansehra Siran Valley. First, the fronds of the selected plant were dried in dark and crushed into fine powder using a conventional grinder. Different solvents (Chloroform, Methyl alcohol, N-hexane, Ethyl acetate, and double distilled water) were used to prepare the plant extracts. 50 grams of fine powder of fronds was added in 400 ml of each solvent and kept in a shaker for 7 days at room temperature. Then F1001 grade filter papers were used to filter out the extracts, then subjected to a rotary evaporator to completely drying out the extracts and till further analysis the dried extracts were stored at 4 °C.

### Sample preparation for nanoparticles synthesis

Hydric extracts *W*. *unigemmata* were to synthesize nanoparticles; 90 ml of distilled water and 0.50968 mg of silver nitrate (AgNO_3_) were added to 10 ml of the hydric extracts of the plant. The mixture was then transferred to the 100 °C heating plate. A change in colour of the mixture (indication of the synthesis of silver nanoparticles) was observed, and then the mixture was transferred to the oven at 65 °C for drying. For antibacterial studies, AgNPs (0.40 mg) was taken, diluted in 10 ml of double distilled water, and analyzed against various bacterial strains [24].

### Characterization

SEM (scanning electron microscopy), EDS (energy dispersive spectroscopy), Fourier transform infrared (FTIR), ultraviolet-visible (UV-VIS) and XRD (XRD) analyses were applied to characterize AgNPs. A UV-vis spectrophotometer was used to analyze the synthesis of silver nanoparticles with an absorption spectrum at a wavelength between 300 and 700 nm. The crystal structure of the silver nanoparticles was detected by XRD. X-ray diffraction was recorded in the 2θ range from 10° to 80°. FTIR analysis was used to confirm the functional group of silver nanoparticles with a spectral range from 4000 to 400 cm^-1^. The shape and size of the AgNPs was determined by using scanning electron microscope. EDS was used to confirm the abundance of elements present in the mixture, especially the silver nanoparticles.

### Phytochemical analyses

Various phytochemicals were determined on the basis of the change in colour which is known as qualitative phytochemical analyses. To determine coumarins, phlorotannins, carotenoids, and alkaloids we followed the standard protocols applied by [25], while tannins, flavonoids, terpenoids, saponins, phenols, glycosides and carbohydrates were analyzed according to the method developed by [26].

Quantitative phytochemical analysis of *W*. *unigemmata* was performed using a spectrophotometer (UV 1900). Different protocols were used to quantify different phytochemicals, for prolines [27], ascorbic acid [28], anthocyanin [29], flavonoids and phenols [30], and sugar content [31] were analyzed accordingly.

### Antibacterial activity

To determine the antibacterial potential of *W*. *unigemmata*, four multidrug resistant bacterial strains (*Escherichia coli* = ATCC-8739, *Klebsiella pneumonia* = ATCC-10031, *Staphylococcus aureus* = ATCC-6538, *and Pseudomonas aeruginosa* = ATCC-1238) were used. For the preparation of serial dilutions of dry extracts, 120 mg/ml of dimethyl sulphoxide (DMSO) was used and the disc diffusion method was applied to determine the antibacterial activity. Blank discs were dipped into DMSO only for use as a negative control, and levofloxacin discs were used as a positive control [32].

### Anticancer activity of *W*. *unigemmata* (ethanolic extract)

Human gastric cancer cell lines (American-type culture collection) AGS (ATCC = CRL-1739) and SGC7901 (ATCC = 7901), and normal gastric epithelial cell line GES-1 were used to analyze the anticancer activity of *W*. *unigemmata*. The AGS cell line was cultured in F12 media containing 10% fetal bovine serum, while RPMI 1640 (Roswell Park Memorial Institute) media supplemented with 10% fetal bovine serum was used to culture the SGC7901 and GES-1 cells. Both cell lines were cultured in a humidified incubator at 37 °C with a constant supply of 5% CO_2_.

### Cell viability assay

The anticancer activity of the ethanolic plant extracts against AGS, SGC7901 and GES-1 cell lines was determined using the cell count kit-8 (CCK-8) assay (Beyotime Biotechnology Co. Ltd., China). Cells in the logarithmic growth phase (24 hours after culture) were subculture in 96-wellplates (5000 cells/well). Cells were treated with different concentrations (08 μg, 16 μg, 32 μg, 62 μg, 125 μg, 250 μg, 500 μg, and 1000 μg) of plant extract; medium without plant extracts and DMSO (solvent) were considered a negative control, while standard anticancer drug (5-FU) was used as a positive control. After 24 hours, culture media were replaced with fresh media (100 ml / well), then 10 μl of CCK-8 was added to each well and incubated at 37 °C for 02 hours. Optical density (OD) was measured at 450 nm using a microplate reader (Bio-Rad Laboratories, Richmond, CA, USA). To ensure the reliability and validity of the results, the experiment was repeated three times for each extract [33] and [34].

### Colony formation assay

Cells in their logarithmic growth phase were harvested and 1x10^3^ cells were seeded in each well of a 6-well plate and incubated at 37 °C in an incubator for 14 days. When the cells started to cluster to form colonies (4 days later), different concentrations (500, 1000, and 2000 μg) of the plant extract were added to each well, while DMSO and media without plant extracts were used as negative controls. To maintain the pH of the medium and smooth cellular growth, the medium was periodically changed. 14 days later, colonies were fixed with 4% paraformaldehyde, stained with crystal violet dye, photographed, and counted.

### Statistics

Statistix 8.1 software was used for data analysis. The mean of the experimental replicates was noted and significant differences between various treatments were determined using ANOVA test. A P-value <0.05 was considered significant.

## Results and discussion

### Qualitative phytochemical analysis *W*. *unigemmata*

We detected various phytochemicals including flavonoids, carotenoids, terpenoids, pholobatanins, alkaloids, saponins, quinines, carbohydrates, glycosides, phenols, tannins, and coumarins in all extracts. Except carotenoids and quinines, all phytochemicals were detected in the methyl alcohol extract, while in N- hexane extracts phlobatanins, saponins, glycosides, carbohydrates, and coumarins were not observed. Similarly, flavonoids, carotenoids, saponins, and carbohydrates were not detected in distilled water extracts (Table 1). Methyl alcohol is a very well-known solvent for analyzing various categories of phytochemicals [35]. Similarly, N-hexane also considered best solvents to identify alkaloids, tannins, sugar, saponins, flavonoids, terpenoids, cardiac glycosides, phenolics, and anthraquinone [25], while distilled water also have potential to extract useful bioactive agents [36].

**Table 1:** Phytochemical constituent of *W*. *unigemmata* in different extracts.

Tests	Chloroform	Ethyl acetate	Methyl alcohol	N-hexane	Distilled water
**Flavonoids**	+++	-	+++	+++	-
**Carotenoids**	+++	+++	-	+++	-
**Terpenoids**	-	++	+++	++	+++
**Phlobatanins**	-	-	++	-	+++
**Alkaloids**	+++	+++	+++	+++	+++
**Saponins**	++	-	+++	-	-
**Quinines**	++	-	-	++	+++
**Carbohydrates**	-	-	++	-	-
**Glycosides**	-	-	++	-	++
**Phenols**	-	-	+++	+++	+++
**Tannins**	++	-	+++	++	+++
**Coumarins**	-	-	+++	-	+++

Key = -, absent; ++, moderately present; +++, strongly present.

The chloroform and ethyl acetate extracts of *W*. *unigemmata* have good extraction of different phytochemicals such as flavonoids, alkaloids and carotenoids were significantly observed, followed by moderate detection of saponins, quinines and tannins. Ayana *et al*. [37] claimed that chloroform is one of good choices of solvents for preparing plant extracts. In contrast, ethyl acetate was shown to have less phytochemical extraction ability [38]. In current study, only carotenoids, terpenoids, and alkaloids were found in the ethyl acetate extracts (Table 1). Phytochemicals that were identified in our study are known to have high medicinal and commercial importance [39], suggesting that *W*. *unigemmata* may have potential medicinal properties.

### Quantifying phytochemicals in *W*. *unigemmata*

**Fig 1:**
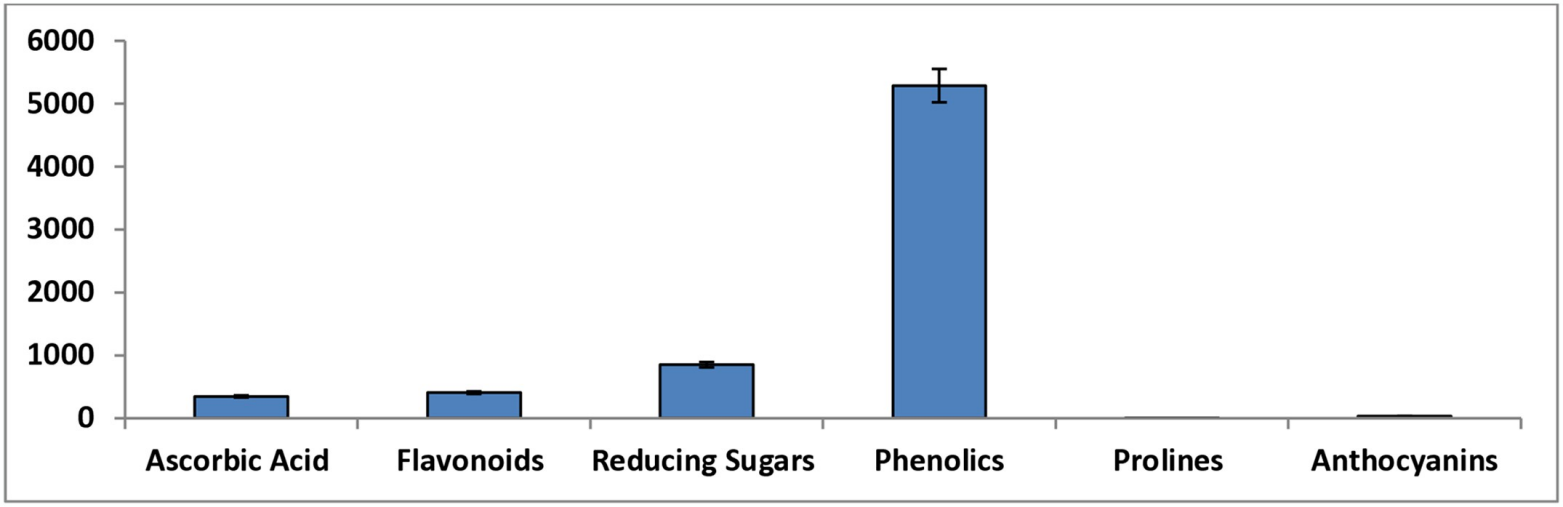
Graphical representation of quantitative phytochemical analysis of *W*. *unigemmata*.

The spectrophotometry revealed a significantly higher amount of phenolics (5289.89 ± 112.67) followed by reducing sugars (851.53 ± 120.15) and flavonoids (408.28 ± 20.26). A significantly lower amount of ascorbic acid (347.64 ± 16.32) was observed, and negligible concentration of anthocyanins (33.32 ± 4.74) and prolines (0.18 ± 0.11) was also determined (Fig 1 and Table 2). Secondary metabolites (phytochemicals) help plants cope with adverse environmental conditions and have medicinal importance for humans [40]. Flavonoids have antimicrobial, anti-inflammatory, anti-cancer and antioxidant properties [41]. Alkaloids are cardioprotective, anti-inflammatory, and anesthetic agents such as morphine is a well-known alkaloid that is used as a strong analgesic drug [42]. Plant phenolics have oxygen-free radical scavenging properties; can be used against oxidation reactions in the body that prevent the development of cardiovascular disorders [43]. Similarly, prolines in the human body act against oxidative stress in different diseases [44]. Anthocyanins are the most important medicinal phytochemicals that can be used in different ailments such as cardiovascular, neurological, cancer, diabetes, and gastric disorders [45]. The qualitative and quantitative phytochemical analysis of *W*. *unigemmata* showed presence of different secondary metabolites, indicating a strong medicinal significance of this plant.

**Table 2:** Quantitative phytochemical analysis of *W*. *unigemmata*.

S.NO	Phytochemicals	Quantities (average ± standard error)	Units
1	Ascorbic Acid	347.64 ± 16.32	mM/g F.WT
2	Flavonoids	408.28 ± 20.26	Mg/g F.WT
3	Reducing Sugars	851.53 ± 120.15	uM/ml F.WT
4	Phenolics	5289.89 ± 112.67	Mg/L F.WT
5	Prolines	0.18 ± 0.11	uM/g F.WT
6	Anthocyanins	33.32 ± 4.74	uM/g F.WT

### Antibacterial properties of *W*. *unigemmata*

**Fig 2:**
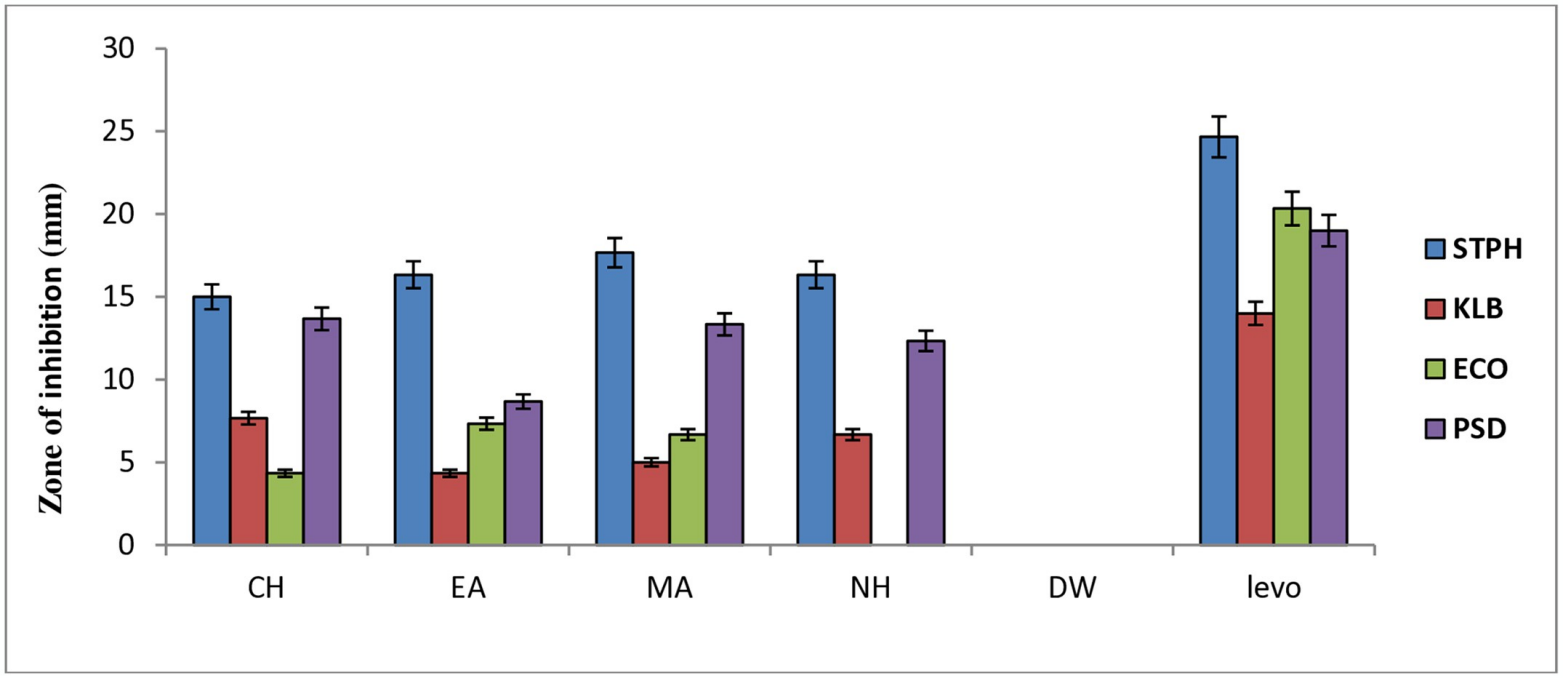
Graphical representation of antibacterial activity of *W*. *unigemmata* (Key- CH = chloroform, EA = ethyl acetate, MA = methyl alcohol, NH = n-hexane, DW = distilled water, LEVO = Levofloxacin, STPH = *S*. *aureus*, KLB = *K*. *pneumoniae*, ECO = *E*. *coli*, PSD = *P*. *aeruginosa)*.

Overall, extracts prepared organic solvents showed better antibacterial activity than the hydric extracts of the *W*. *unigemmata* (Fig 2). We speculate that antibacterial components of the selected plant are non-polar in nature so could not be extracted in the distilled water; thus, showing negative results against selected bacterial strains. Previously, hydric extract of the areca nut fruit showed no activity *against S*. *aureus*, *E*. *coli*, and *S*. *enterica*, while organic solvent extracts (ethanolic and methanolic) were highly active [46]. However, some plants contain water soluble active compounds [47].

**Fig 3:**
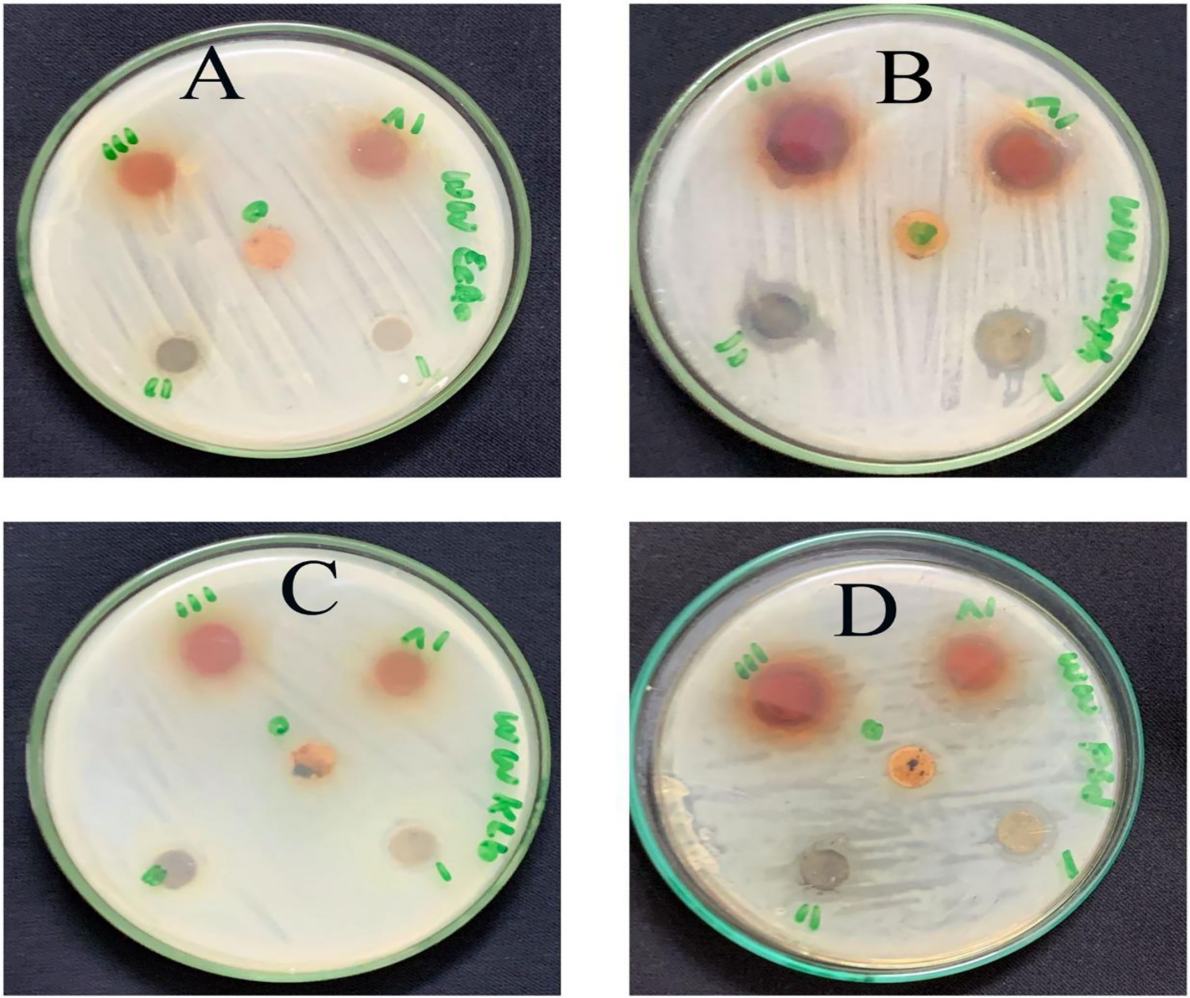
Antibacterial activity of *W*. *unigemmata* (i = chloroform, ii = N-hexane, iii = ethyl acetate, IV = methyl alcohol, 0 = distill water) A = *E*. *coli*, B = *S*. *aureus*, C = *K*. *pneumoniae*, D = *P*. *aeruginosa*.

The chloroform extract showed a maximum inhibition zone against *S*. *aureus* (15±0.57) followed by *P*. *aeruginosa* (13.66±0.88). Chloroform extract was found to be less effective against *K*. *pneumoniae* and *E*. *coli*. Similarly, the ethyl acetate extract of *W*. *unigemmata* showed a higher resistance zone against *S*. *aurous* (16.33±0.33) followed by *P*. *aeruginosa* (8.66±4.33), while this extract produces statistically non-significant inhibitory zones against *E*. *coli* and *K*. *pneumoniae*. The highest zone of inhibition was observed against *S*. *aureus* (17.66±0.33) in methyl alcohol extract followed by *P*. *aeruginosa* (13.33±1.66) while this extract was less active against *E*. *coli* and *K*. *pneumoniae*. In case of N-hexane extract, the growth of *S*. *aureus* and *P*. *aeruginosa was* significantly retarded with inhibitory zones (16.33±0.33) and (12.33±0.88) respectively, while this extract showed less activity against *K*. *pneumoniae* and inactive against *E*. *coli*; all activities were compared with levofloxacin and DMSO (Table 3). The activity shown by organic solvent extracts might be due to the non-polar nature of the antibacterial compounds of the *W*. *unigemmata*. Antibacterial and antifungal activity of organic solvent extracts of *D*. *viride*, prepared in methanol, ethanol and chloroform showed better inhibitory response against pathogenic fungus and bacteria [48]. Our findings also revealed that *S*. *aureus* and *P*. *aeruginosa* were the most sensitive strains, while *K*. *pneumoniae* and *E*. *coli* were the most resistant strains to plant extracts. Previously, *P*. *aeruginosa* and S. *aureus* are considered the most sensitive strains to *C*. *longa* rhizome extract [49] and *A*. *calamus* rhizome extracts [50]. *K*. *pneumoniae* and *E*. *coli* showed resistance to organic extracts of *W*. *unigemmata*, we hypothesized that both bacterial strains are Gram negative, having an extra membrane of lipopolysaccharide on their cell walls that prevents entry of antibacterial compounds. Similarly, essential oil extracts of *M*. *cajuputi* are less effective against *K*. *pneumoniae* and *E*. *coli* [51], while methanolic leaf extracts of *S*. *anquetilia* are highly active against *K*. *pneumoniae*, *E*. *coli*, *S*. *aureus*, and *P*. *aeruginosa* [52] (Fig 3).

**Table 3:** Antibacterial activity of different solvents extracts of *W*. *unigemmata* (Zone of inhibition (mm) ± Standard Error means).

S.NO	Strains	CH	EA	MA	NH	DW	LEVO
1	*Staphylococcus aureus*	15±0.57	16.33±0.33	17.66±0.33	16.33±0.33	00±00	24.66±0.88
2	*Klebsiella pneumoniae*	7.66±3.92	4.33±4.33	5±5.00	6.66±3.38	00±00	14±1.15
3	*Escherichia coli*	4.33±4.33	7.33±3.84	6.66±3.33	0±0.00	00±00	20.33±0.88
4	*Pseudomonas aeruginosa*	13.66±0.88	8.66±4.33	13.33±1.66	12.33±0.88	00±00	19±0.57

Key- CH = chloroform, EA = ethyl acetate, MA = methyl alcohol, NH = n-hexane, DW = distilled water, LEVO = Levofloxacin.

### Antibacterial activity of silver nanoparticles synthesized form *W*. *unigemmata*

**Fig 4:**
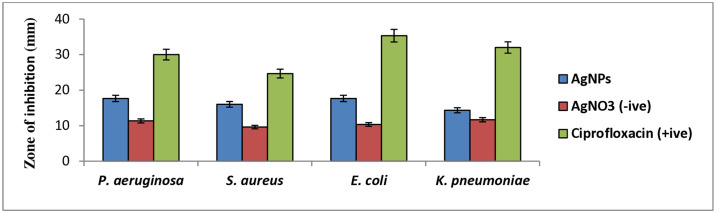
Graphical representation of antibacterial activity of greenly synthesized silver nanoparticles (AgNPs) from aqueous extract of *W*. *unigemmata*.

The aqueous extracts of *W*. *unigemmata* were used to synthesize silver nanoparticles (AgNPs), and their antibacterial potential against selected MDR bacterial strains was determined. A previous study reported that these bacterial strains are resistant to different classes of antibiotics [53]. Greenly synthesized silver nanoparticles show an efficient antibacterial potential as shown in Fig 3. Significantly higher growth inhibition was observed against *E*. *coli* and *P*. *aeruginosa* with a similar zone of inhibition measured as (17.66 ± 1.85), followed by *S*. *aureus* (16.00 ± 1.73), while (14.33 ± 1.20) zone of inhibition (14.33 1.20) was observed against *K*. *pneumoniae*. The inhibitory zones produced by the negative control (AgNO_3_) were subtracted from the zones produced by greenly synthesized AgNPs (Fig 4 and Table 4). Greenly synthesized silver nanoparticles showed significant activity against *K*. *pneumoniae*, *E*. *coli*, and *S*. *aureus* [54]. Similarly, AgNPs synthesized from an aqueous extract of *S*. *officinalis* produced better inhibitory effects against *E*. *cloacae*, *E*. *coli*, *K*. *pneumoniae*, *S*. *typhimurium*, and *P*. *aeruginosa* [55]. Our findings indicate that all of the selected bacterial strains were sensitive to AgNPs synthesized from *W*. *unigemmata*; we believe that bioactive components of plant attach with the surface of silver nanoparticles and efficiently penetrate the bacteria.

**Table 4:** Antibacterial activity of greenly synthesized silver nanoparticles (Zone of inhibition (mm) ± Standard Error means).

S.NO	Bacterial strains	Zone of inhibition (AgNPs)	AgNO3 (-ive)	Ciprofloxacin (+ive)
01	*P*. *aeruginosa*	17.66 ± 1.85	11.33 ± 0.88	30 ± 1.15
02	*S*. *aureus*	16.00 ± 1.73	9.6 ± 0.70	24.66 ± 1.45
03	*E*. *coli*	17.66 ± 1.85	10.33 ± 0.90	35.33 ± 1.50
04	*K*. *pneumoniae*	14.33 ± 1.20	11.66 ± 0.88	32 ± 1.15

### Characterization of AgNPs

**Fig 5:**
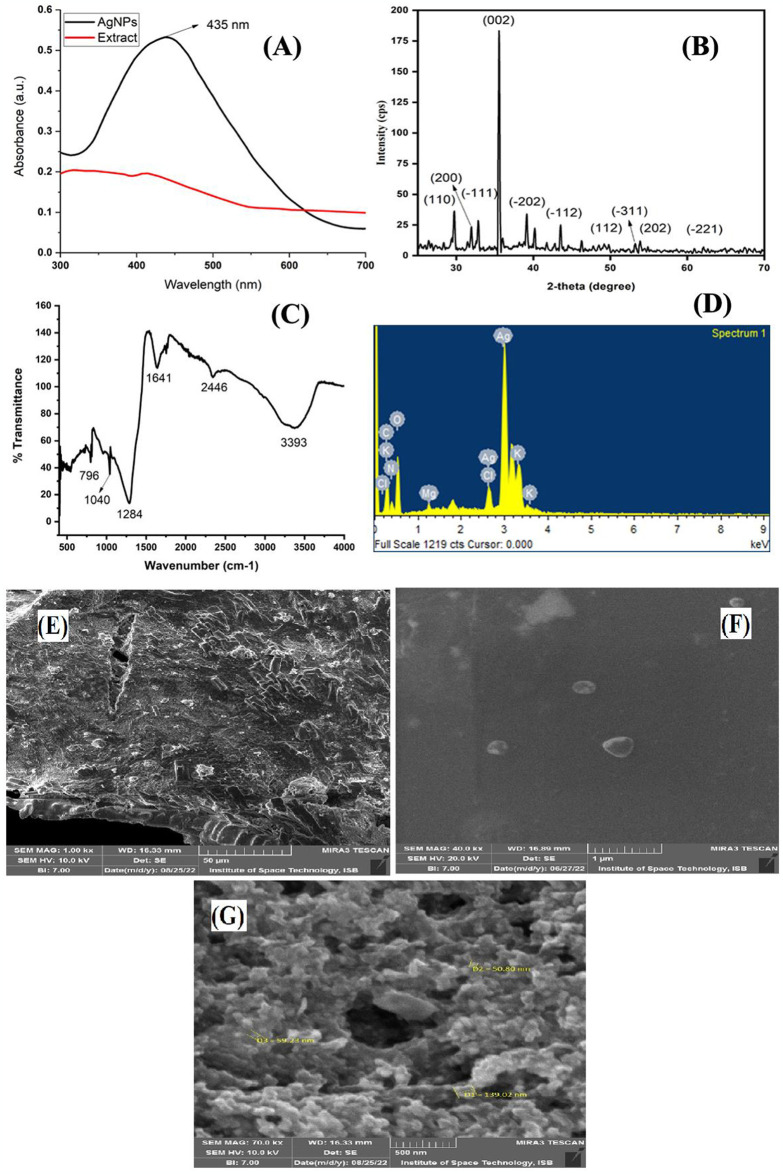
Characterization of *W*. *unigemmata* silver nanoparticles (A = UV-VIS, B = XRD, C = FTIR, D = EDS, E, F, G = SEM).

Various characterization techniques used for the detection of greenly synthesized silver nanoparticles using aqueous frond extract of *W*. *unigemmata*. First, UV-VIS spectra were used to confirm the reduction of Ag^+^ to Ag^0^ (Fig 5 (A)). The absorption spectra of UV-VIS showed a peak at 435nm, clearly indicating the formation of silver nanoparticles. This UV-vis band is due to surface plasmon resonance, which confirmed the synthesis of silver nanoparticles. The phytochemicals interacting with synthesized nanoparticles interfere with surface plasmon resonance (SPR) absorbance, which produces absorption peaks of UV-vis spectroscopy. Different plant extracts have different phytoconstituents that interfere with the surface plasmon resonance absorbance differently and produce a variable range of UV-Vis band spectra of different wavelengths [56]. A similar absorption range (425–475 nm) for AgNPs reported by [57] is due to surface plasmon resonance, which indicates fast bio-reduction of silver nanoparticles.

### XRD analysis of AgNPs

Fig 5(B) shows the peaks in the XRD patterns. The crystalline nature of greenly synthesized silver nanoparticles was confirmed by XRD. One major sharp peak (002) was observed at 35.5°, which determined the face-centered cubic structure of AgNPs. Other smaller peaks (110), (200), (-111), (-202), (-112), (112), (-311), (202) and (-221) are detected at 29.71°, 31.91°, 32.91°, 39.18°, 43.51°, 49.48°, 53.24°, 54.92°, and 62.17° respectively, represents the presence of bioactive compounds of W. unigemmata extract, which reduce and stabilize silver nanoparticles. These peaks are consistent with the ICSD reference code: 01-075-0969. The specific crystallographic planes observed are well matched with known patterns for AgNPs, which supports the claimed composition of the synthesized nanomaterials.

### FTIR analysis of AgNPs

Similarly, the surface nature of silver nanoparticles was determined by FTIR Fig 5(C)). Prominent bands of absorption were observed around 3393, 2446, 1641, 1284, 1040, and 796 cm^-1^. These peaks denote O-H, N-H (ammonium ions), C = C, C-O (ether), C-O (alcohol), and aromatic C-H, respectively. These bands represent the stretching vibration of compounds like flavonoids, terpenoids, and phenolics present in the extract of *W*. *unigemmata*, and these compounds are responsible for the capping of AgNPs and stabilizing their structures. FTIR banding patterns confirm that greenly synthesized AgNPs were capped with phytoconstituents such as phenolics, flavonoids, and terpenoids were present in our plant extracts. The presence of these phytochemicals is confirmed during the qualitative and quantitative phytochemical analyses of our plant. These biological compounds are responsible for the stabilization of AgNPs structures and prevent the aggregation of greenly synthesized silver nanoparticles. [58], claim that the O-H and C = O groups provide the information that AgNO_3_ is reduced to Ag (0). These two stretching vibrations are prominent in our greenly synthesized AgNPs, which confirms the presence of reduced silver nanoparticles. Our findings are consistent with the literature [59], and describe the FTIR banding patterns of AgNPs synthesized from *H*. *graveolens* extract. They observed prominent peaks at 3382, 2927, 1513, 1417, 1035, 820, and 606 cm^-1^ which represents the stretching vibrations of O-H, CO, C = O, CO, O-H, CO, C-O, and C-X, respectively.

### EDS analysis for of AgNPs

EDS analysis also confirms the reduction of AgNO_3_ to Ag (0), as shown in (Fig 5(D)) with a clear large Ag peak. Other smaller peaks represent the element based on plant extract (Mg, Cl, O, K, and N). The findings of the current study similar to the results of [58], they claim that silver nanoparticles synthesized from extracts of *R*. *luteolus* and *P*. *leucomelas* show that EDS peaks other than Ag are part of phytochemicals of plant extract which act as a capping agent for synthesized AgNPs. Similar results are also presented by [60]; they also observed smaller peaks (O, C, K, Na, Ca) other than the Ag peak in the EDS of AgNPs from the butterfly pea plant.

### Size and shape of AgNPs

The shape and size of greenly synthesized silver nanoparticles were determined by SEM as shown in (Fig 5E–5G). SEM analysis revealed the oval and triangular shape of AgNPs and the average size of biosynthetically prepared silver nanoparticles was 82 nm. The size of AgNPs by SEM analyses ranges from 50–100 nm [61]. The silver nanoparticles synthesized from different plants show different shapes, i.e., round, spherical, hexagonal, and triangular such as AgNPs prepared from *Aloe vera* extract have a triangular shape [62].

### Effect of *W*. *unigemmata* on proliferation of gastric cancer cells

**Fig 6:**
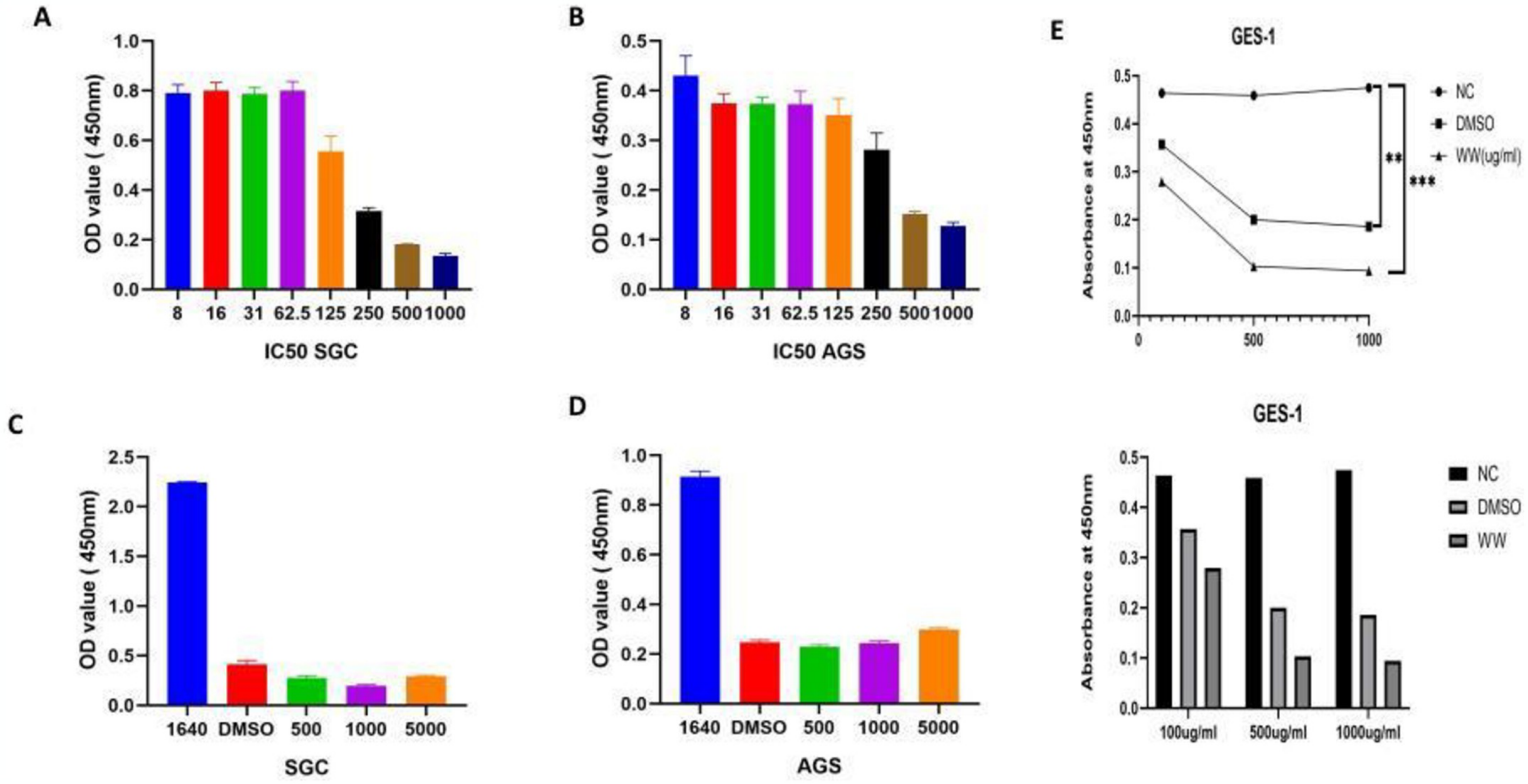
Graphical representation of the anticancer activity of *W*. *unigemmata*, A = SGC 7901 cell line, B = AGS cell line, C = proliferation assay against AGS cell line, D = proliferation assay against SGC cell line, E = CCk8 assay for normal gastric epithelial cells (GES1).

Different concentrations of ethanolic extracts were used to analyze the anticancer activity of *W*. *unigemmata*. Initially, gastric cancer cell lines were subjected to IC_50_ analysis, indicating that the higher concentration of 500–1000 g of plant extract significantly resists the growth of gastric cancer cell lines. In contrast, cancer cell lines (AGS and SGC7901) show a significant increase in growth at lower concentrations of plant extract (Fig 6A and 6B). These findings might be due to the fact that plants contain bioactive compounds such as glycosides, lignins, flavonoids, coumarins, etc., that may support cellular functions. We compared these findings with normal gastric epithelial cells GES-1 and found that at higher concentration *W*. *unigemmata* significantly inhibit the cell growth of both cancer and normal cells (Fig 6(E)). At a higher concentration of the extract, the amount of biologically active phytochemicals become elevated, which causes the death of cancerous cells and low concentrations are unable to kill cancer cells due to deficiency of these compounds in the extract. Significant cell growth arrest was observed in the SGC7901 and GES-1 cell lines at a plant extract concentration of 500–5000 mg compared to the control, while these plant extract concentrations were inactive against the AGS cell line (Fig 6C and 6D).

**Fig 7:**
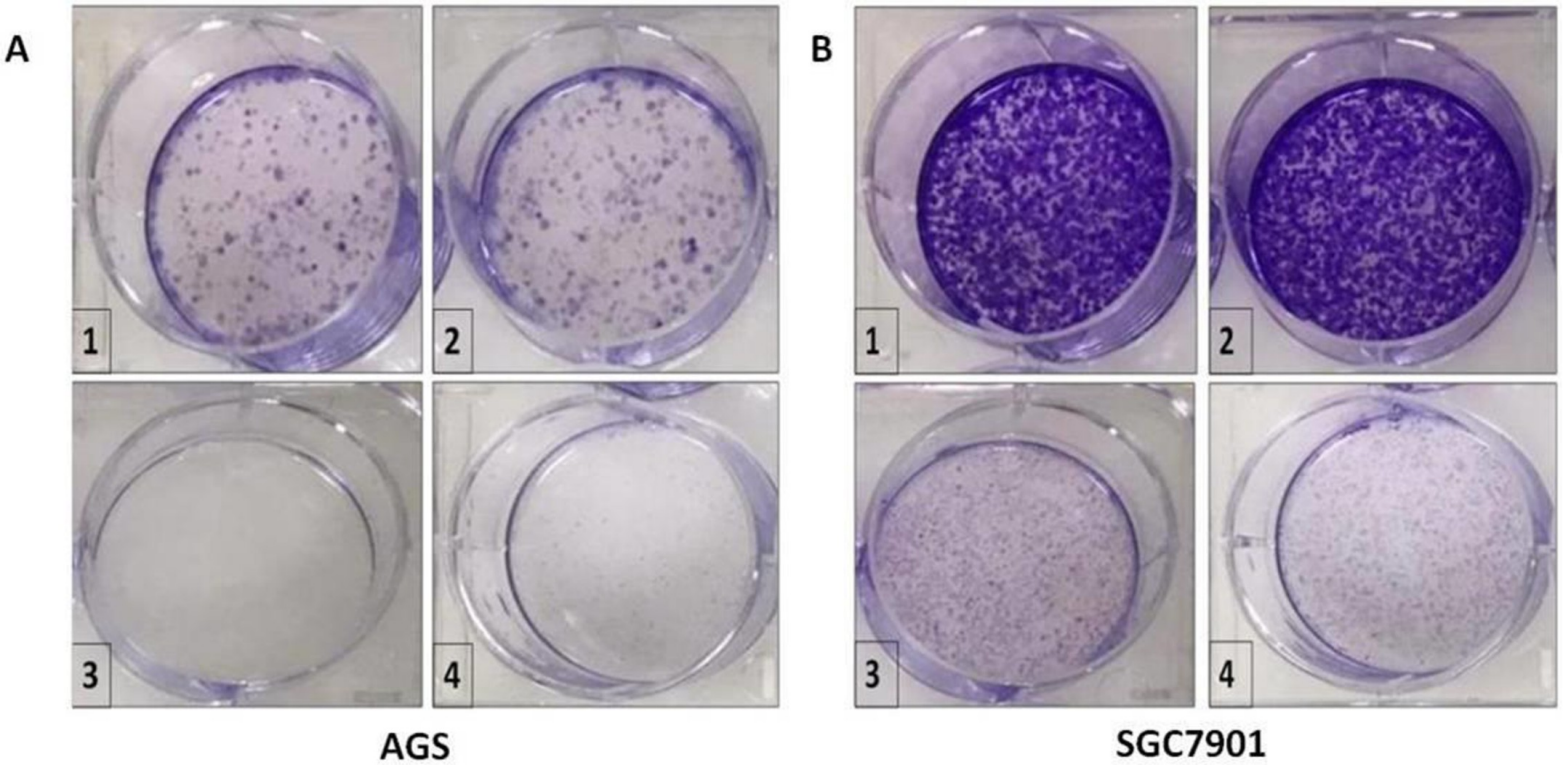
Colony formation assay. A) colony formation assay against AGS cell line (1 = cancer cell with normal media, 2 = cancer cells with DMSO, 3 = cancer cells with 500ug plant extract, 4 = cancer cells with 2000ug plant extract), B), colony formation assay against SGC7901 cell line (1 = cancer cell with normal media, 2 = cancer cells with DMSO, 3 = cancer cells with 500ug plant extract, 4 = cancer cells with 2000ug plant extract).

Furthermore, we performed a colony formation assay (Fig 7A and 7B), which showed that 500 and 2000 mg extracts of *W*. *unigemmata* had a greater growth inhibition of the AGS, SGC7901 cell line compared to the negative control. Using natural products as anticancer agent has gained a high reputation recently, as Ohiagu *et al*. [63] also emphasized on the role of bioactive compounds against cancer. Several plants showed strong anticancer activity against different cancer cell lines colo205, Km12, A498, U031, HEP3B, SKHEP, MG63, and MG63.3 [64], and breast cancer cell lines (MCF-7 and MDA-MB-231) [65]. A plant called *S*. *officinale* even at lower concentration significantly inhibited the growth of the breast cancer cell line (MCF7) [66]. Le *et al*. [67] synthesize silver nanoparticles using *Ardisia gigantiflia* leaves extract for anticancer activities using human gastric cancer cell lines, their results suggest that Arg-AgNPs caused cell cycle arrest at the G0/G1 phase and suppressed cell migration. N Hao *et al*. [68] also synthesized silver nanoparticles using solution plasma method in the presence of P. *trimera* extract. They also observed strong anticancer activity for the AGS gastric cancer cell line. Our study has some limitations; although, *W*. *unigemmata* showed significant antimicrobial effects, also produced cytotoxicity against human gastric cancer and normal cells. We were not able to analyze the underlying mechanisms of antimicrobial and cytotoxic effects. We did not purify the compound through which *W*. *unigemmata* exert biological activity.

## Conclusion

Based on current results, we believe that *W*. *Unigemmata* enriched with bioactive phytochemicals and thus can be used as an additional drug against MDR bacterial strains and but still require further validations for supplementing it for gastric cancer due to high cytotoxic effect. Furthermore, these biomolecules covering the AgNPs may enhance their biological activities, therefore, can be used in the management of different microbial infections.
